# Importance of secondary screening with clinical isolates for anti-leishmania drug discovery

**DOI:** 10.1038/s41598-018-30040-5

**Published:** 2018-08-06

**Authors:** Aya Hefnawy, Juan Cantizani, Imanol Peña, Pilar Manzano, Suman Rijal, Jean-Claude Dujardin, Geraldine De Muylder, Julio Martin

**Affiliations:** 1 Department of Biomedical sciences, Institute of Tropical Medicine, Antwerp, Belgium; 2 Diseases of the developing world, GlaxoSmithKline, Tres cantos, Spain; 3 BP Koirala Institute of Health Sciences, Dharan, Nepal; 4 Department of Biomedical Sciences, Faculty of Pharmaceutical, Biomedical and Veterinary Sciences, University of Antwerp, Antwerp, Belgium

## Abstract

The growing drug resistance (DR) raises major concerns for the control of visceral leishmaniasis (VL), a neglected disease lethal in 95 percent of the cases if left untreated. Resistance has rendered antimonials (SSG) obsolete in the Indian Sub-Continent (ISC) and the first miltefosine-resistant *Leishmania donovani* were isolated. New chemotherapeutic options are needed and novel compounds are being identified by high-throughput screening (HTS). HTS is generally performed with old laboratory strains such as *LdBOB* and we aimed here to validate the activity of selected compounds against recent clinical isolates. In this academic/industrial collaboration, 130 compounds from the GSK “Leishbox” were screened against one SSG-sensitive and one SSG-resistant strain of *L. donovani* recently isolated from ISC patients, using an intracellular assay of *L. donovani*-infected THP1-derived macrophages. We showed that only 45% of the compounds were active in both clinical isolates and *LdBOB*. There were also different compound efficiencies linked to the SSG susceptibility background of the strains. In addition, our results suggested that the differential susceptibility profiles were chemical series-dependent. In conclusion, we demonstrate the potential value of including clinical isolates (as well as resistant strains) in the HTS progression cascade.

## Introduction

Leishmaniasis is a neglected tropical disease caused by parasites of the genus *Leishmania*^1^. It occurs in several parts of the world under different clinical forms, the most severe being visceral leishmaniasis (VL), also known as kala-azar; a systemic illness that is lethal if left untreated. Every year, 200,000–400,000 new cases of VL happen, due to parasites of the *L. donovani* complex, essentially in India, Nepal, Bangladesh, Sudan, Ethiopia and Brazil, with a mortality estimated at 10%^1^. In the Indian sub-continent (ISC) a regional program aiming for the elimination of VL (Kala-Azar elimination program or KAEP) is currently running, based on two major pillars, vector control and chemotherapy^2^.

Currently, there are only few therapeutic options for leishmaniasis and they have various drawbacks^3^. Pentavalent antimonials (SSG) were used for decades, but they are limited by toxicity and treatment failure (TF) primarily caused by resistant parasites. This led to the progressive withdrawal of SSG in the Indian Sub-Continent (ISC) since 2005^4^. At that time, SSG was replaced by Miltefosine (MIL), the first oral treatment available for leishmaniasis^5^. However the use of MIL is being threatened by the alarming rise of TF in the ISC^6^ and recently, the first MIL-resistant strains were detected in the field^7^. The development of a liposomal form of Amphotericin B (Ambisome) has reduced its toxicity but its availability to patients is restricted by high costs^8^. Preferential pricing agreement increased access to Ambisome and a single-dose treatment is now used as the first line treatment in the ISC^9^. Combination therapies are used as an alternative, but this strategy might be questioned, when one of the composing drug is starting to fail (like MIL); in addition, experimental work has shown that *L. donovani* resistant to combined drugs could easily be selected *in vitro*^10^. Currently there are no other drugs available, and new compounds, formulations or combinations are urgently needed.

High-throughput screening (HTS) is an efficient way of identifying active compounds^11,12^ able to eliminate the parasite without affecting the host. Whole-cell phenotypic HTS is used for anti-leishmania drug discovery because of the low number of molecular targets well characterized and validated^13^. The parasite life stage used for screening is of utmost importance and intracellular amastigotes have been recognized as the ideal model^14^. High content image analysis can be incorporated in the screen to quantify changes in cellular phenotype, which is called high content screening (HCS). The first HCS/HTS using intracellular amastigotes infecting human macrophages was reported in 2010^15^ and since then, improvements of HTS protocols have been continuously carried out to reach a good balance of practicality, reproducibility and biological relevance. *L. donovani* BOB strain (*LdBOB*, MHOM/SD/62/1S-CL2D) has been the strain of choice for many HTS campaigns^16–18^. It is a clone of Ld1S2D, an East African strain isolated in 1962, that has been maintained in laboratories over the world for decades^19^. It is of utmost importance to check whether compounds, which are active against laboratory-adapted strains like LdBOB, will be equally active against clinical isolates that may differ genetically and phenotypically (virulence, drug resistance…). This approach, albeit well established in drug discovery against other pathogens, is not common in the *Leishmania* community and was recently recommended^3^.

Recently, GlaxoSmithKline (GSK) has carried out a HTS on a diversity set of 1.8 million compounds through whole-cell phenotypic assays against wild type and eGFP-engineered *LdBOB*. This generated the “Leishbox” comprising 192 compounds with anti-leishmania activity^18^. We aimed here to explore the relevance of clinical isolates in the further validation of these compounds. For this proof-of-principle study, we selected two clinical isolates of *L. donovani* from our ISC collection, respectively sensitive and resistant to SSG and we included them in the screening assay. We show here that 45% of the tested compounds of the Leishbox were pan-active, while 55% were inactive in one or the other clinical isolates. These results demonstrate the importance of a secondary screening with clinical isolates, in support to the selection process and profiling of the hits that can proceed further in the drug discovery pipeline.

## Results

### Optimization of the HCA for clinical isolates

**Figure 1:**
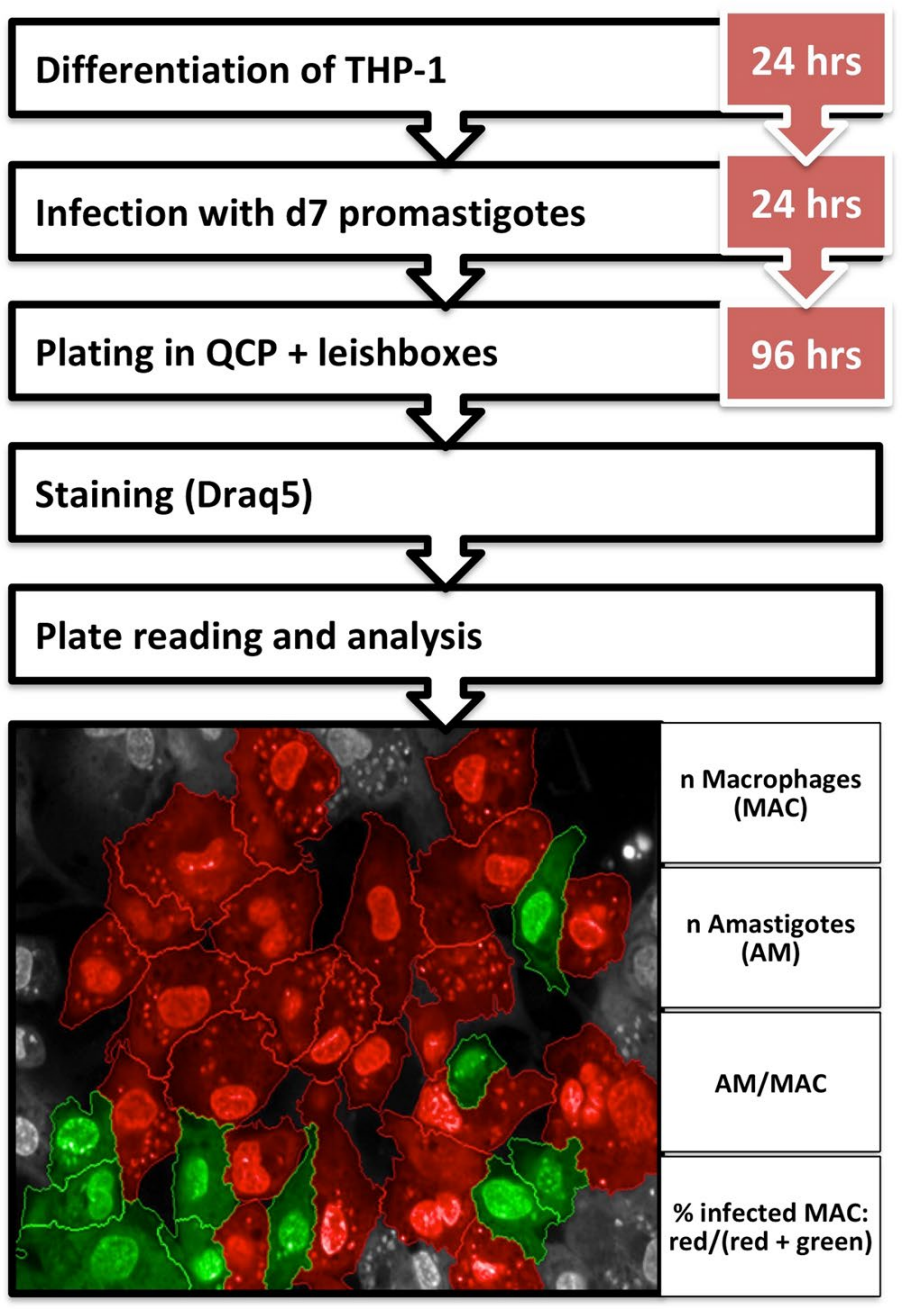
Experimental setup and analysis parameters of the InMAC assay. THP-1 cells are differentiated for 24 hrs followed by o/n infection with d7 promastigotes. Infected THP-1 cells are plated and incubated with the “Leishbox” compounds and controls for 96 hrs. Finally plates are stained with Draq5, read and analysed. Images were taken by Opera at 40x magnification. The infected cells are shown in red while the non-infected ones are shown in green.

The InMac assay developed by GSK was modified by the incorporation of horse serum which increased the robustness and the predictive potential of the assay by reducing the amount of extracellular promastigotes remaining after infection^17^. Moreover, it avoided the reinfection and increased the replication rate of intracellular amastigotes^17^. The assay was further optimized for the clinical isolates LdBPK_282 and LdBPK_275 (procedure described in Fig. 1).

**Figure 2:**
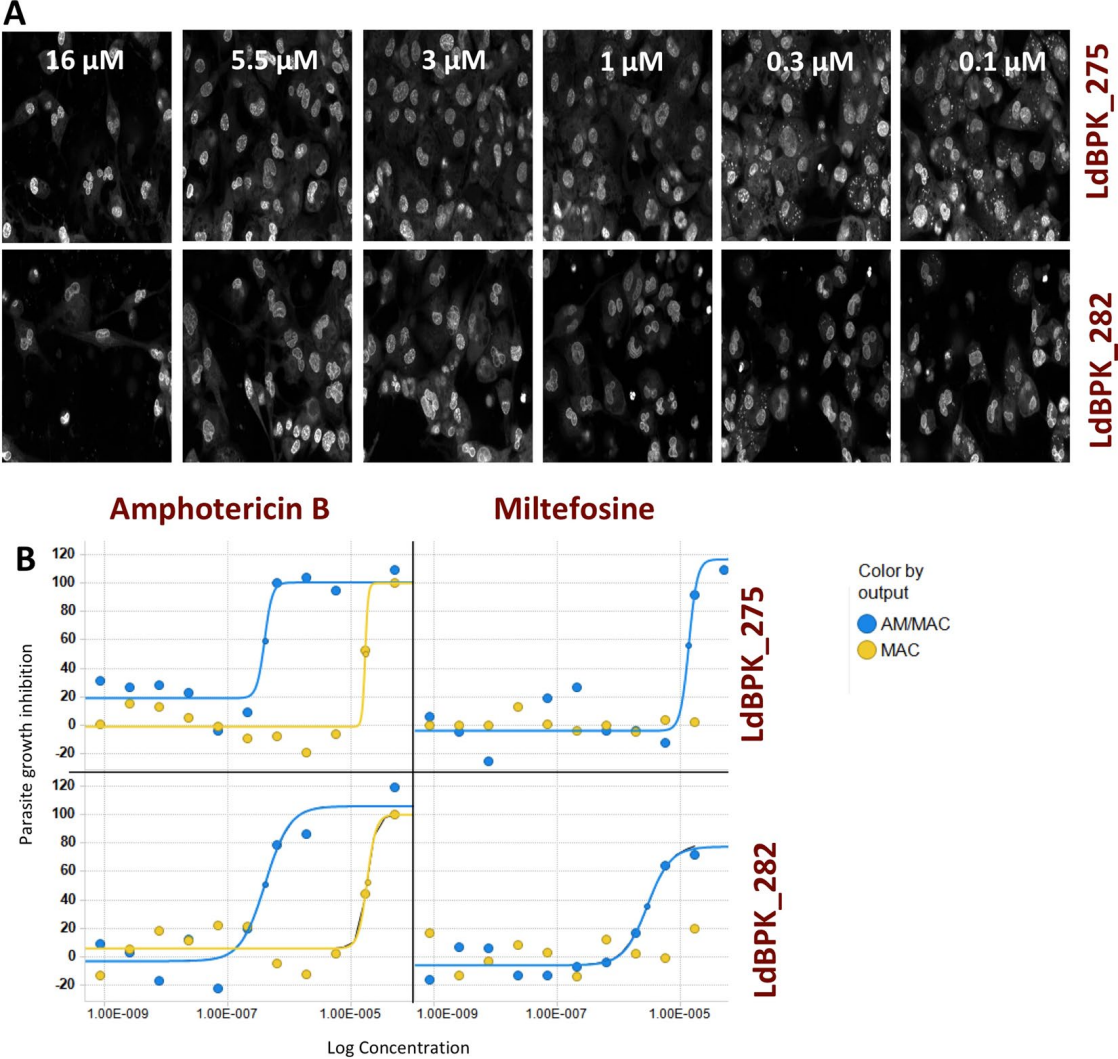
Quality control of the InMac Assay. (**A**) A 40x magnification image from Opera of 96 h Amphotericin B dose response curve (0.1 μM to 16 μM) of THP-1 infected with LdBPK_275 in the upper panel and LdBPK_282 in the lower panel at MOI 30, cells are stained with Draq5. (**B**) Dose response curves for Amphotericin B and Miltefosine for LdBPK_275 in the upper panel and LdBPK_282 in the lower panel; the concentration is represented on the X-axis (M) and the response on the Y-axis. The amastigote/macrophage (AM/MAC) readout is represented in blue and macrophage (MAC) readout in yellow.

The infection with clinical isolates provided various challenges. A multiplicity of infection (MOI) of 30 was needed to achieve a satisfactory infection level (compared to an MOI of 10 in case of *LdBOB*). Infections reached a maximum of 55% of infected cells in the case of LdBPK_275 and 44% in the case of LdBPK_282 compared to almost 90% of infected cells with *LdBOB*. There was also a slight variability in the number of amastigotes per macrophage with an average value of 3.2 for LdBPK_275, 2.7 for LdBPK_282 and 3 for *LdBOB*. Despite these challenges, the results obtained with drugs used in clinical practice were consistent with the previously reported values, hereby validating the sensitivity of the system (Fig. 2). Amphotericin B showed activity against both clinical isolates. The measured IC_50_ was 0.25 μM in LdBPK_282 (pIC_50_ 6.6) and 0.316 μM in LdBPK_275 (pIC_50_ 6.5). In literature^20^ the IC_50_ reported for amphotericin B against *L. donovani* was 0.1 to 0.4 μM (pIC_50_ 7 to 6.4, respectively). For MIL, the obtained IC_50_ values were 2.95 μM in LdBPK_282 (pIC_50_ 5.53) and 3.58 μM in LdBPK_275 (pIC_50_ 5.45) while the values reported in literature were 0.9 to 4.3 μM (pIC_50_ 6.04 to 5.36, respectively)^20^. In addition to these two gold standards, 22 control compounds were included for each run, these compounds belong to internal drug discovery programs and have different modes of action and potency ranges. The correlation between historic *LdBOB* data and the 2 clinical isolates was excellent. For this assay and based on these compounds, Minimum Discriminatory Difference (MDD)^21^ was 0.36, which gives confidence in the quality of the replicates. Regarding the reproducibility and the quality of the assay, even though the Z’ values were low for both strains, the automated imaging assay for wild type clinical isolates had enough statistical significance to evaluate the activity of compounds in dose-response. Finally, screening of the “Leishbox” was run in duplicate obtaining excellent correlations for the 2 copies. Calculated MSD (minimum significant difference) as the average of 2*SD of all the compounds tested is 0.4, a value which is within the acceptable range reported by other authors^22^.

### Activity of “Leishbox” compounds against L. donovani clinical isolates

**Figure 3:**
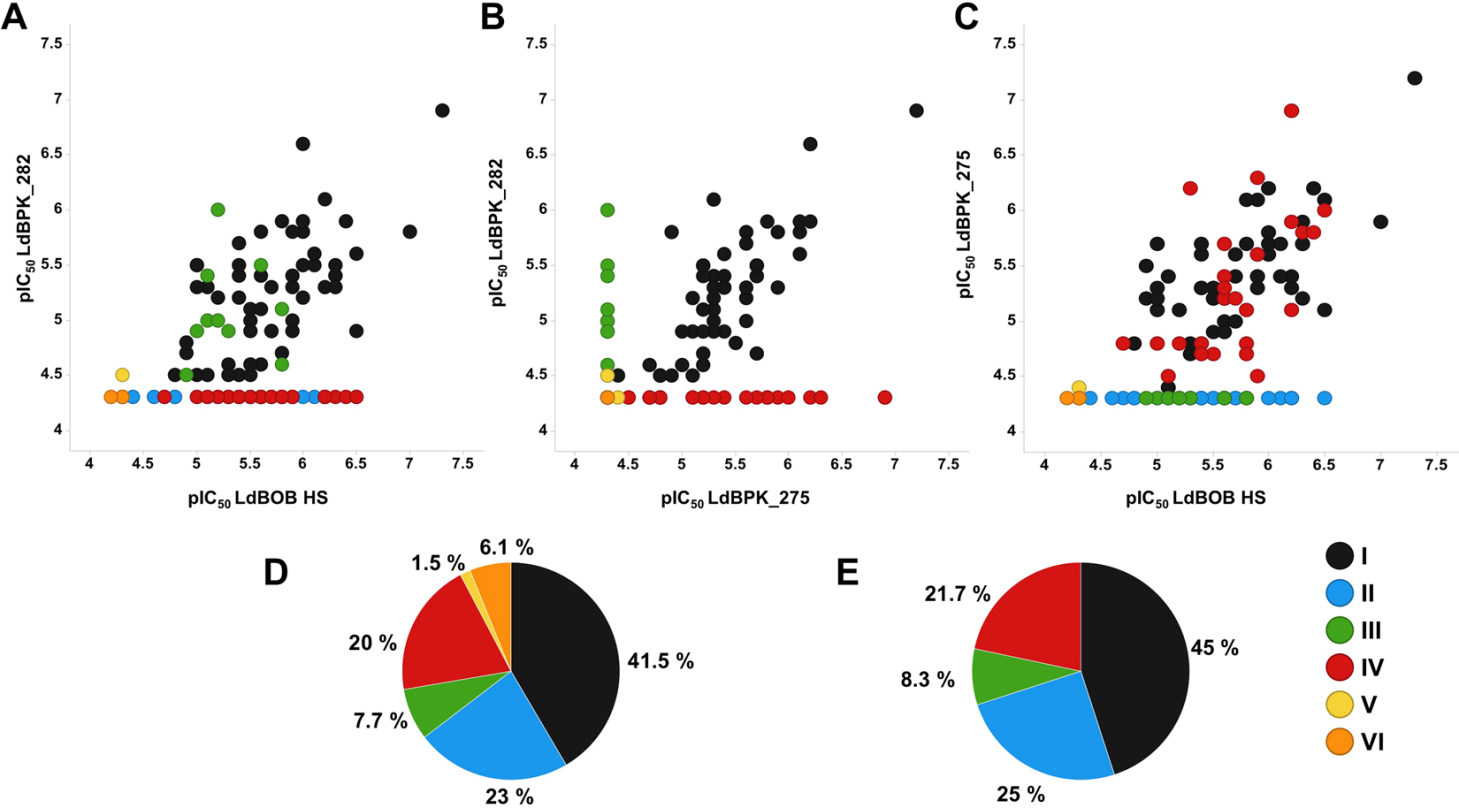
Differential efficacy of compounds of the leishbox on the different *LD* strains. A-C Correlation of the pIC_50_ values calculated from the AM/MAC readout, inactive compounds are represented with pIC_50_ of 4.3 (corresponding to an IC_50_ of 50 μM). (**A**) SSG-S vs *LdBOB*. (**B**) SSG-S vs SSG-R. (**C**) SSG-R vs *LdBOB*. Compounds are colour coded based on their categories (I-VI). (**D**,**E**) The percentage of compounds per category. (**D**) Representation of the six categories of the 130 compounds. (**E**) Representation of the 120 compounds active against *LdBOB* (categories I-IV).

A set of 130 compounds from the “Leishbox” was selected based on availability (Table 1). The compounds’ activity against LdBPK_282 and LdBPK_275 was tested and compared to their documented values against *LdBOB* (values obtained after incorporation of horse serum in the assay as described elsewhere^23^). The results shown are the pIC_50_ values as calculated from the amastigote per macrophage readout (AM/MAC). Ten compounds lost their activity against *LdBOB* after the assay modification and addition of horse serum. The remaining 120 compounds were then classified into 4 categories, from I to IV (Fig. 3). Compounds that show activity in the three strains of *L. donovani* fall in category I (pan-active compounds: 45%). Compounds that lose activity in both clinical isolates fall in category II (25%). While those that show activity in *LdBOB* and LdBPK_282 (SSG-S) only are in category III (8.33%), and those active only in *LdBOB* and LdBPK_275 (SSG-R) are in category IV (21.66%). Among the 10 compounds that lost activity with *LdBOB*, 2 showed activity against one of the clinical isolates: they formed category V. The final set of compounds (8) did not show activity against any of the tested strains (category VI).

**Table 1:** AMMAC pIC50 values.

ChEMBL ID	ldBPK_275	ldBPK_282	*LdBOB*	Compound category
TCMDC-124508	5,5	4,8	4,9	I
TCMDC-125826	4,5	4,3	5,9	IV
TCMDC-134026	4,8	4,3	5,2	IV
TCMDC-142704	4,3	4,3	5,4	II
TCMDC-142900	5,4	5,6	6,1	I
TCMDC-143072	4,3	4,3	5,6	II
TCMDC-143075	4,4	4,5	5,1	I
TCMDC-143077	4,3	4,3	6,2	II
TCMDC-143078	4,3	4,3	5,2	II
TCMDC-143090	5	4,6	5,6	I
TCMDC-143091	5,3	5,2	5,4	I
TCMDC-143094	5,2	4,6	5,5	I
TCMDC-143095	5,2	5,1	5,6	I
TCMDC-143096	5,2	4,3	5,7	IV
TCMDC-143098	4,3	4,3	4,7	II
TCMDC-143099	4,3	4,3	4,3	VI
TCMDC-143101	5,7	5,4	6,3	I
TCMDC-143106	5	4,9	5,7	I
TCMDC-143115	4,3	4,9	5	III
TCMDC-143117	4,3	4,3	5,6	II
TCMDC-143119	4,3	4,3	4,3	VI
TCMDC-143124	4,5	4,3	5,1	IV
TCMDC-143129	5,7	5,5	5,4	I
TCMDC-143133	6,1	5,6	6,5	I
TCMDC-143136	4,3	4,3	5,7	II
TCMDC-143144	5,4	4,3	5,6	IV
TCMDC-143145	5,2	4,3	5,6	IV
TCMDC-143163	4,3	4,3	6,1	II
TCMDC-143164	5,6	5	5,9	I
TCMDC-143165	5,3	5,3	5	I
TCMDC-143166	4,3	4,3	5,3	II
TCMDC-143168	6	4,3	6,5	IV
TCMDC-143169	4,7	4,3	5,5	IV
TCMDC-143170	5,3	5	5,5	I
TCMDC-143171	5,6	4,3	5,9	IV
TCMDC-143174	5,2	5,4	5,6	I
TCMDC-143175	4,3	4,3	5,4	II
TCMDC-143181	4,3	4,3	5,6	II
TCMDC-143188	4,3	4,3	5,1	II
TCMDC-143196	5,4	5,3	5,1	I
TCMDC-143197	4,3	4,3	5,4	II
TCMDC-143201	4,3	4,3	5	II
TCMDC-143202	4,3	4,3	4,6	II
TCMDC-143208	4,3	4,5	4,9	III
TCMDC-143211	5,4	5,3	5,9	I
TCMDC-143212	5,8	4,3	6,4	IV
TCMDC-143213	5,9	5,8	7	I
TCMDC-143214	5,4	5,3	6,2	I
TCMDC-143215	5,8	4,3	6,3	IV
TCMDC-143216	5,9	5,3	6,3	I
TCMDC-143218	5,9	4,3	6,2	IV
TCMDC-143236	5,4	4,9	5,7	I
TCMDC-143238	5,7	5,5	6,1	I
TCMDC-143239	4,3	6	5,2	III
TCMDC-143245	4,8	4,3	4,7	IV
TCMDC-143246	4,4	4,3	4,3	V
TCMDC-143249	5,2	4,7	4,9	I
TCMDC-143255	4,3	4,3	4,4	II
TCMDC-143261	4,9	4,5	5,5	I
TCMDC-143266	4,3	4,3	4,8	II
TCMDC-143269	4,3	4,9	5,3	III
TCMDC-143271	4,3	4,3	5,7	II
TCMDC-143274	5,3	5,4	5,4	I
TCMDC-143277	5,1	4,5	5	I
TCMDC-143278	4,8	4,3	5	IV
TCMDC-143280	6,2	4,3	5,3	IV
TCMDC-143281	6,9	4,3	6,2	IV
TCMDC-143285	4,9	5,8	5,6	I
TCMDC-143287	4,3	4,3	4,3	VI
TCMDC-143295	7,2	6,9	7,3	I
TCMDC-143305	5,6	5,2	6	I
TCMDC-143306	4,3	5,4	5,1	III
TCMDC-143315	5,6	5,3	5,7	I
TCMDC-143327	6,2	6,6	6	I
TCMDC-143344	5,6	5,7	5,4	I
TCMDC-143347	6,1	5,8	5,9	I
TCMDC-143348	4,3	4,3	4,8	II
TCMDC-143349	4,3	4,3	4,2	VI
TCMDC-143350	4,8	4,3	5,8	IV
TCMDC-143351	6,1	5,9	5,8	I
TCMDC-143353	4,3	5	5,2	III
TCMDC-143355	4,3	4,3	4,3	VI
TCMDC-143358	5,1	4,9	6,5	I
TCMDC-143367	4,3	4,3	4,3	VI
TCMDC-143375	5,1	5,2	5,2	I
TCMDC-143383	4,7	4,3	5,8	IV
TCMDC-143388	4,3	4,3	5,8	II
TCMDC-143391	4,3	4,3	6	II
TCMDC-143397	4,7	4,3	5,4	IV
TCMDC-143398	5,4	5,4	5,9	I
TCMDC-143404	5,7	4,7	5,8	I
TCMDC-143406	4,3	4,3	6,5	II
TCMDC-143418	4,8	4,3	5,4	IV
TCMDC-143419	4,3	4,3	5,3	II
TCMDC-143431	4,8	4,5	5,3	I
TCMDC-143442	4,3	4,3	5,4	II
TCMDC-143443	4,3	5,5	5,6	III
TCMDC-143448	4,3	4,3	5,5	II
TCMDC-143459	5,2	5,5	6,3	I
TCMDC-143473	4,8	4,5	5,4	I
TCMDC-143478	5,7	4,3	5,6	IV
TCMDC-143487	5,3	6,1	6,2	I
TCMDC-143503	5,7	5,5	6	I
TCMDC-143509	5,1	4,3	5,8	IV
TCMDC-143514	4,3	4,3	5,3	II
TCMDC-143521	4,3	5,1	5,8	III
TCMDC-143522	5,2	4,9	5,5	I
TCMDC-143523	4,3	4,5	4,3	V
TCMDC-143524	6,2	5,9	6,4	I
TCMDC-143532	4,3	4,3	4,3	VI
TCMDC-143534	5,3	5,1	5,5	I
TCMDC-143558	5,7	5,4	6,3	I
TCMDC-143563	4,3	4,3	5,5	II
TCMDC-143566	4,8	4,5	4,8	I
TCMDC-143568	6,3	4,3	5,9	IV
TCMDC-143570	5,3	4,9	5,9	I
TCMDC-143571	5,7	5,5	5	I
TCMDC-143573	4,3	5	5,1	III
TCMDC-143577	5,2	5,5	5	I
TCMDC-143584	5,1	4,3	6,2	IV
TCMDC-143586	5,8	5,9	6	I
TCMDC-143591	5,3	4,3	5,6	IV
TCMDC-143594	4,3	4,3	4,3	VI
TCMDC-143603	6,9	4,3	6,2	IV
TCMDC-143607	4,3	4,3	5,6	II
TCMDC-143621	4,3	4,3	5,6	II
TCMDC-143628	4,3	4,3	6,2	II
TCMDC-143630	5,6	5,8	6	I
TCMDC-143633	4,7	4,6	5,3	I
TCMDC-143639	4,3	4,6	5,8	III

### Chemical analysis of the “Leishbox” compounds results

**Figure 4:**
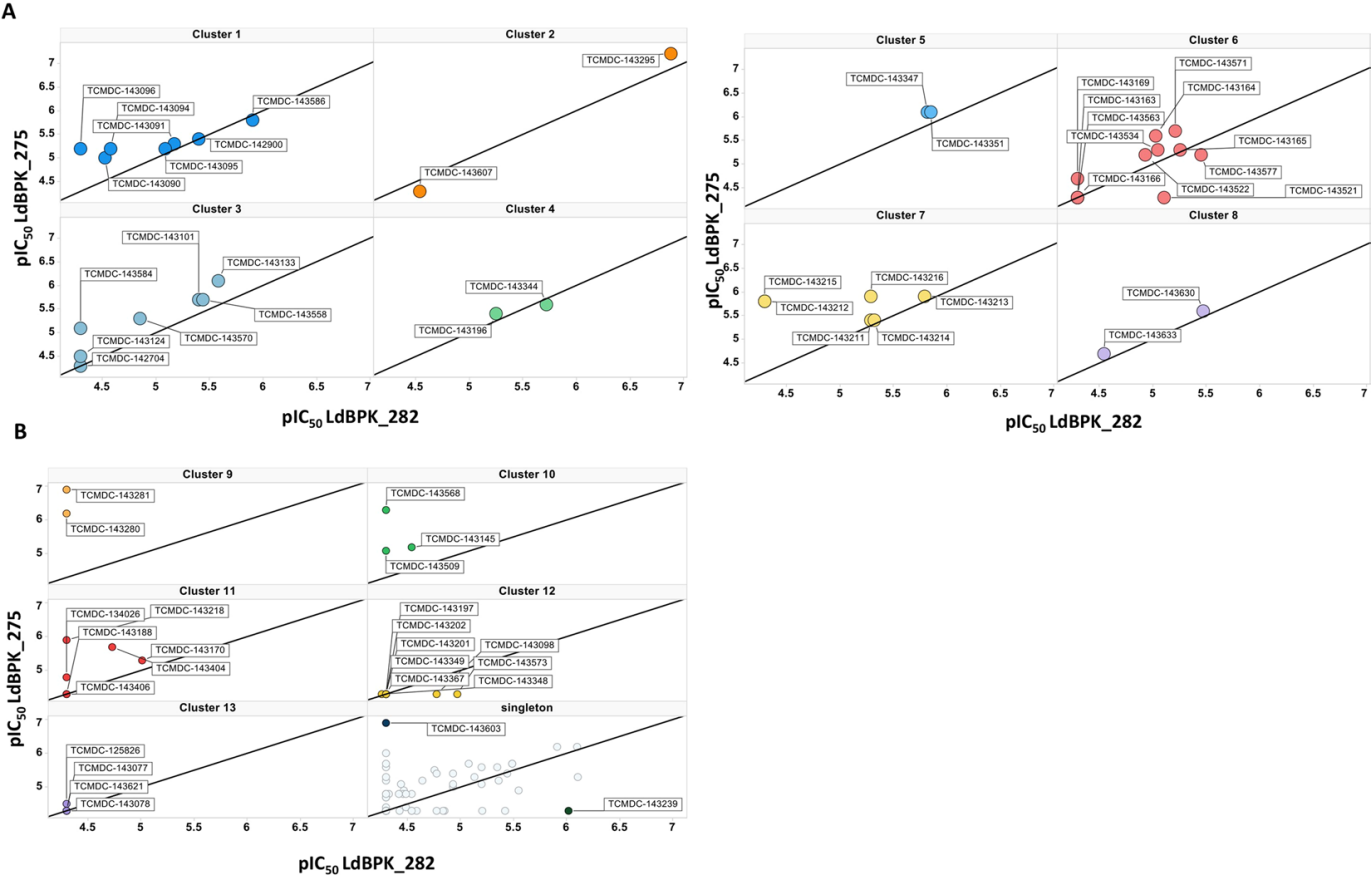
Chemical clustering of tested compounds. On the X axis pIC_50_ of LdBPK_282 and on the Y axis pIC_50_ of LdBPK_275. Compounds from the “Leishbox” were visually grouped on the basis of common substructures resulting in 13 clusters with at least 2 members and 68 singletons. For each cluster at least 50% of the compounds have the same biological behaviour. (**A**) Pan-active compounds, cluster 1 to 8. (**B**) Cluster 9, 10 and 11 includes compounds selective to SSG-R and Cluster 12 and 13 were inactive. A plot with 68 singletons was included to illustrate the data. The two most active singletons were commented in the text and marked and labelled in the figure.

The results of the screening were analysed in function of the chemical structure of the compounds and their hypothetical targets (Table 2 and Fig. 4). Compounds were grouped on the basis of common substructures using Helium as the cheminformatics toolkit that provides search by substructure within a table (the “Leishbox” in this case) through SMILES data, which resulted in the delineation of 13 chemical clusters with at least two members and 68 singletons. Interestingly, compounds of the same chemical cluster generally showed similar activity versus the strains used and at least 50% of the members of each cluster presented the same activity profile (Fig. 4). Clusters 1 to 8 included pan-active compounds (category I), clusters 9, 10 and 11 included compounds which are more active against LdBPK_275 and *LdBOB* than against LdBPK_282 (category IV), clusters 12 and 13 included the compounds that lost activity in the assay with clinical isolates (category II). For category III (active only on LdBPK_282 and *LdBOB*

**Table 2:** Chemical clustering of the tested compounds.

Chemical series	No. of compounds	Compounds	Target class associated	LD275/LD282 classification*
	7	TCMDC-143090		Panactive
		TCMDC-143091		
		TCMDC-143094		
		TCMDC-143095		
		TCMDC-143096	Antiviral	
		TCMDC-142900	Other Enzyme	
		TCMDC-143586		
	2	TCMDC-143295		Panactive
		TCMDC-143607		
	7	TCMDC-143101	Cytochrome	Panactive
		TCMDC-142704	TB	
		TCMDC-143124		
		TCMDC-143133		
		TCMDC-143558		
		TCMDC-143570		
		TCMDC-143584	Other Enzyme	
	2	TCMDC-143196		Panactive
		TCMDC-143344		
	2	TCMDC-143347	Cytochrome, Other Enzyme	Panactive
		TCMDC-143351	Cytochrome, Other Enzyme	
	11	TCMDC-143163		Panactive
		TCMDC-143164	Other Enzyme	
		TCMDC-143165		
		TCMDC-143166	Other Enzyme	
		TCMDC-143169		
		TCMDC-143521	Other Enzyme	
		TCMDC-143522		
		TCMDC-143534	Other Enzyme	
		TCMDC-143563	Other Enzyme	
		TCMDC-143571		
		TCMDC-143577		
	6	TCMDC-143211		Panactive
		TCMDC-143212		
		TCMDC-143213		
		TCMDC-143214		
		TCMDC-143215		
		TCMDC-143216		
	2	TCMDC-143630	kinase	Panactive
		TCMDC-143633		
	2	TCMDC-143280	Kinase, Cytochrome, Other Enzyme	275 selective
		TCMDC-143281	Kinase	
	3	TCMDC-143145	Protease	275 selective
		TCMDC-143509	Protease, cytochrome	
		TCMDC-143568		
	6	TCMDC-143170	Other Enzyme	275 selective
		TCMDC-143188		
		TCMDC-143218	TB	
		TCMDC-134026		
		TCMDC-143404	Kinase	
		TCMDC-143406	Cytochrome	
		TCMDC-143603	Cytochrome	275 selective
		TCMDC-143239	Kinase, Antiviral, Other Enzyme	282 selective
	8	TCMDC-143348		Inactive
		TCMDC-143349		
		TCMDC-143367	Kinase, Other Enzyme	
		TCMDC-143098		
		TCMDC-143197		
		TCMDC-143201		
		TCMDC-143202		
		TCMDC-143573		
	4	TCMDC-143077		Inactive
		TCMDC-143078		
		TCMDC-125826		
		TCMDC-143621		

*LD275/LD282 classification representing at least 50% of the cluster members.

), compound TCMDC-143521 was found in cluster 6 and compound TCMDC-143573 was found in cluster 12. The most active compound in category III was the singleton TCMDC-143239.

## Discussion

In a recent opinion paper^3^, we highlighted that most HTS were done using a single reference strain like *LdBOB*, a strain isolated in Sudan in 1962 and since then maintained for decades in several laboratories. This strain is particularly adapted for HTS, because of its ability to grow and infect macrophages *in vitro*. However, considering (i) its status of ‘laboratory-adapted’ strain, (ii) the big genomic differences existing between *L. donovani* from East Africa and from the Indian sub-continent^24^ and (iii) the reported differences in treatment efficacy between East Africa and ISC^25^, it is currently unknown whether/how *LdBOB* is representative of clinical isolates and parasites from different geographical regions, in terms of drug susceptibility. Therefore, it was recommended to add clinical isolates to the screening cascade following the primary HTS process, including drug-resistant ones^3^.

The present study is the first experimental validation of this recommendation, with a specific emphasis on the ISC. The two Nepalese strains used here were isolated from VL patients in 2003, maintained for a maximum of 45 passages *in vitro* and showing different susceptibility to SSG. Using clinical isolates has generally been avoided in HTS because of the difficulty of achieving high quality infections *in vitro*. Several factors can reduce the quality of the infection including the excess of extracellular promastigotes, which can interfere with the efficacy of the analysis, especially for compounds that are active only on intracellular stages. However addition of horse serum (HS) as recommended by the recent study^17^ proved to be a good strategy. Another factor could be the lower *in vitro* infectivity of clinical isolates of *L. donovani*; in the present study, a higher MOI was needed, but it still resulted in a lower percentage of infected cells (around 50%) than in the case of *LdBOB* (around 90%). However, these infection levels were sufficient to obtain reliable results and our quality controls validated the sensitivity of the system in terms of the pharmacology response. Moreover, the obtained data for activity of MIL and Amphotericin B fully agreed with the values reported in literature.

We reported here that out of 120 compounds active on *LdBOB* (i) 45% were also active on the two clinical isolates, independently of their SSG susceptibility (pan-active), (ii) 25% lost their activity on the two clinical isolates, (iii) while 21.7 and 8.3% were only active on the SSG-R and the SSG-S clinical isolate, respectively. Only 2 compounds were active on one clinical isolate and inactive on *LdBOB*, hereby supporting the further use of *LdBOB* for primary screening. However, our results strongly confirm the interest of introducing clinical isolates at a second stage of screening. The obvious next question now concerns the implications of these results in an R&D context: should pan-active compounds be prioritized in the pipeline and what should be done with the compounds inactive on one or more clinical isolates?

To answer this question a wide panel of strains, which provides a true representation of the actual clinical setting in the world will be essential. For visceral leishmaniasis, and taking into account genomic and phenotypic diversity, clinical isolates of the following species/variants should be taken into consideration: *L. donovani* from ISC, *L. donovani* from East Africa and *L. infantum*. For each of them, wild-type as well as strains resistant to drugs currently in use should be included (ideally clinically resistant; if not experimentally selected). This will rapidly give a significant number of lines and the next question will be to make these compatible with the secondary screening or could be part of a final validation step. Finally, such sets of well-characterized strains with regards to the genome and the metabolome, should be gathered and distributed to potential users.

Pan activity should be prioritized and, if there is specific susceptibility of some strains, the compounds should at least cover all strains of a particular geographical population. Since development costs are huge, “personalized” or segmented medicines for VL are not feasible so there is not enough value in a drug candidate that covers only a segment of population at risk.

At the current stage, it is impossible to know if the absence of activity of a fifth of the compounds against two clinical isolates of *L. donovani* from the ISC is due to genetic and phenotypic differences between East African and ISC parasites or to the difference between a laboratory-adapted strain and clinical isolates. Further work including recent clinical *L. donovani* isolates from East Africa is required, but most likely the two factors must play a role. The differences observed here between the two ISC isolates also deserve a deeper attention. Differences were expected, considering the major functional differences existing between populations represented by both strains, beyond their SSG-susceptibility profiles: differences in (i) capacity for protection against oxidative stress, (ii) fluidity of plasma membrane, (iii) metabolite survival kit and (iv) capacity to up-regulate host IL-10 and to over-express multidrug-resistant protein 1 of the macrophage^26–28^. However, the higher number of active compounds on the SSG-R isolate vs. the SSG-S is surprising, SSG-R parasites being reported to be more virulent and fit, even in the absence of the drug^29^. At the contrary, the molecular adaptations towards SSG, observed in LdBPK_275 could have made it more sensitive to other drugs. Only 8.3% of the compounds were inactive on the SSG-R strain. As above, further work is required including more SSG-R and SSG-S isolates and those resistant to other drugs used in the field, in order to further document the impact of resistance when screening for new compounds.

An analysis of the data available in the public domain has allowed us to relate the common substructure in chemical clusters with biological features. This result contributed to further validate the specificity of our data. Clusters 1 to 8 included pan active compounds (category I). The first cluster contained eight bi-pyridinyl compounds whose target has been hypothetically identified as a gene related with Nicotinate and nicotinamide metabolism^18^. The scaffold of pyrimido-pyrimidines has been previously described as DHFR inhibitor mainly for compounds that contain 2,4-diamino substitution in pyrimidine ring (cluster 2)^30^. The compounds sharing a *N*-(4-(pyridin-2-yl)thiazol-2-yl) amide in their structure (cluster 3) showed activity in both strains. This chemical feature was previously identified as antimycobacterial^31^. The compounds of cluster 6 contain a nitro-furane in their structure and 7 out of the 11 compounds were pan active. Nitro drugs are well known to treat parasitic diseases (e.g. nifurtimox, benznidazole, fexinidazole) upon selective activation by parasite nitroreductases^32^. Clusters 9, 10 and 11 include compounds, which are more active against LdBPK_275 *vs*. LdBPK_282. Six members of cluster 11 share the *N*-methyl-4-(1*H*-pyrazol-1-yl)-1,3,5-triazin-2-amine core that has been previously described as an anti-plasmodium agent^33^. Singleton TCMDC-143603 also shows a higher activity against LdBPK_275 *vs*. LdBPK_282 and it contains a primaquine-like structure, a known antimalarial drug. Cluster 12 and 13 included the compounds that have lost their activity in the assay with clinical isolates. The loss of activity of cluster 12, dioxolanes compounds, has been reported in a previous assay with *L. donovani* using horse serum as supplement in the testing culture media^23^. However, in the case of cluster 13 (2-(pyridin-2-yl) pyrimidin-4-amines) the absence of activity could be related to the use of clinical isolates instead of laboratory strains. Overall, these results suggest that the differential susceptibility profile against *Leishmania* strains is more related with the mode of action, thus chemical series-dependent, than with the particular physicochemical features of the compounds.

In conclusion this study resulted from an inter-institutional, academic-industrial collaboration, aiming at improving the drug discovery process for anti-leishmanials. Based on our results, including a multi-strain panel is of utmost importance for the improvement of the preclinical stage of anti-leishmania drug discovery. In particular, in the context of VL chemotherapy, screening should involve recent clinical isolates of the *L. donovani* complex, with different susceptibility profiles to existing drugs and from different geographical areas. Noteworthy, a similar problem arose in the context of diagnostics of VL. The rK39-based ICT rapid diagnostic test provided high sensitivity and specificity in the ISC and Brazil. Yet it showed to be less sensitive and less specific in sub-Saharan Africa^34,35^. One explanation was the extensive kinesin genetic diversity identified between strains from East Africa and ISC^36^. The use of clinical isolates for the validation of HTS can increase the predictive potential of the *in vitro* assays. It also can eliminate the chance of TF by knowing if the drugs are species-specific, which can facilitate identifying the target population that will benefit from the new anti-leishmania drugs released if they do proceed to further production stages. Diversity of *Leishmania* is a reality, which must be taken into account in the quest for new tools for diseases control.

## Methods

### Parasites and mammalian cell cultures

THP-1 cells; (human monocytic leukemia) (ATCC-TIB-202) were provided by the GSK- Biological Reagents and Assay Development Department (BRAD, Stevenage, UK). The THP-1 cells were maintained in RPMI media (Life Technologies) supplemented with 1.25 mM pyruvate (Life Technologies), 2.5 mM glutamine (Life Technologies), and 25 mM HEPES (Life Technologies), and 10% heat-inactivated fetal bovine serum (FBS) (Gibco). Two *L. donovani* cloned strains*:* MHOM/NP/03/BPK282/0 clone 4 (SSG-Sensitive/LdBPK_282) passage number R40/17 (meaning 40 passages since original isolation from patient, including 17 passages since cloning) and MHOM/NP/03/BPK275/0 clone 18 (SSG-Resistant/LdBPK_275) passage number R45/14 were used in the study. The strains were isolated from Nepalese patients with confirmed VL, and cryo-preserved at the Institute of Tropical Medicine, in Antwerp, Belgium. Promastigotes were maintained at 26 °C in M199 medium supplemented with 20% (v/v) heat- inactivated fetal calf serum.

### Intra-macrophage *L. donovani* assay

384-well Assay plates (CellCarrier Ultra, PerkinElmer) were prepared by adding 250 nL of compound to each well with an Echo 555 (Labcyte). For potency determination, eleven-point, one in three dilution curves were generated with a top concentration of 50 μM. The assay was adapted from de Rycker *et al*.^37^ and Peña *et al*.^18^. THP-1 cells were grown in CELLMASTER roller bottles (Greiner cat. #680048) for 72 hrs. The cells were monitored using an optical microscope and counted using a CASY cell counter. Differentiation was carried out in 225 cm^3^ T-FLASK in the presence of 30 nM of phorbol 12-myristate 13-acetate (PMA) (Sigma- Aldrich). Following incubation for 24 h at 37 °C, 5% CO_2_, cell differentiation was visually confirmed using an optical microscope. Cells were washed twice using cell culture media. Cells were infected using day-7 promastigotes of LdBPK_282 or LdBPK_275 at a multiplicity of infection (MOI) of 30 in the T-FLASK containing differentiated THP-1 cells. Each T- FLASK was incubated overnight and the remaining parasites were removed by washing three times with sterile DPBS. The infected cells were harvested by treatment with 0.05% (w/v) trypsin plus 0.48 mM EDTA for 5 mins and an aliquot was fixed and counted in a CASY cell counter. A cell preparation with a final concentration of 1.6*10^5^ cells/mL was prepared in assay media consisting of RPMI (InvitrogenTM), supplemented with 2% heat-inactivated horse serum (HS)^17^ (Gibco) and 25 mM sodium bicarbonate (InvitrogenTM) containing 30 nM of PMA. Infected cells were plated onto assay plates containing the compounds (3000 cells/well, 50 μL) using a Multidrop Combi dispenser and incubated for 96 hrs at 37 °C. Plates were then fixed and stained by the addition of 50 μl of a solution containing 8% formaldehyde and 4 μM Draq5 DNA dye (BioStatus, UK) per well. The complete methodology is summarized in Fig. 1.

### Image and data analysis

Images were taken with a HCS system (Opera QEHS, PerkinElmer) with a 40x objective, five fields per well, for a final number of 300 THP-1 cells per well. Automated image analysis was performed with an image analysis algorithm developed on Acapella high-content imaging and analysis software (PerkinElmer). The THP-1 cell count (MAC) and the average number of amastigotes per macrophage (AM/MAC) were calculated for each well, using the building blocks included in the analysis program. Briefly, using Draq5 signal, the host nuclei and cytoplasm were detected. Inside the cytoplasm spots were selected and filtered using morphology and texture properties identifying the amastigotes. Dose response curves were created and the pIC50 (−log IC_50_ – in molar units) was calculated. Data were normalized to percent of biological response by using positive (i.e. highest response achieved by non infected cells, R_Ctrl2_) or negative (i.e. lowest response achieved in presence of DMSO, R_Ctrl1_) controls by using equation (1):

1 $$ \% Response=\frac{|{R}_{Ctrl1}-{R}_{x}|}{|{R}_{Ctrl1}-{R}_{Ctrl2}|}\cdot 100$$

where Rx is the assay response measured for the compound X. R_Ctrl1_ and R_Ctrl2_ are calculated as the average of replicates in the same microtiter plate where the compound X is tested. pIC50 values were obtained using the ActivityBase XE (IDBS, Guilford, Surrey, UK) nonlinear regression function in the full curve analysis bundle.

Minimum discriminatory difference (MDD) was calculated using equation (2) as follows:

2 $$MDD=\frac{2.3\times S}{\surd N}$$

where S is the pooled variance of the pIC50 determinations of the repeated compounds and N are the replication level of the assay.

### Biosafety

Experimental procedures with *L. donovani* were carried out following standard operating procedures in compliance with biosafety level 3 (BSL3) regulations. THP-1 cells were treated according to GSK policies for the manipulation of human biological samples.

### Data availability

All data generated or analysed during this study are included in this published article.
